# Characteristics and treatment results of patients with gastroenteropancreatic neuroendocrine tumors in a tertiary care centre

**DOI:** 10.1186/s12902-023-01326-1

**Published:** 2023-04-07

**Authors:** Shouki Bazarbashi, Mohamed Aseafan, Tasnim Elgazzar, Maha Alkhayat, Abdulrahman Alghabban, Marwa I. Abdelgawad, Bader Alshamsan, Aisha Alshibany, Tusneem Elhassan, Ali Aljubran, Ahmed Alzahrani, Hindi Alhindi, Hussein Raef

**Affiliations:** 1 Section of Medical Oncology, Oncology Center, King Faisal Specialist Hospital and Research Center, Riyadh, 11211 Saudi Arabia; 2 College of Medicine, Alfaisal University, Riyadh, 11533 Saudi Arabia; 3 Section of Medical Oncology, Department of Internal Medicine, Security Forces Hospital Program, Riyadh, 11481 Saudi Arabia; 4 Clinical Oncology Department, Assiut University, Asyut, Egypt; 5 Department of Medicine, College of Medicine, Qassim University, Qassim, Saudi Arabia; 6 Oncology Center, King Faisal Specialist Hospital and Research Center, Riyadh, 11211 Saudi Arabia; 7 Department of Pathology and Laboratory Medicine, King Faisal Specialist Hospital and Research Center, Riyadh, 11211 Saudi Arabia; 8 Section of Endocrinology, Department of Internal Medicine, King Faisal Specialist Hospital and Research Center, Riyadh, 11211 Saudi Arabia

**Keywords:** Neuroendocrine tumor, Gastroenteropancreatic, GEP-NET, Survival

## Abstract

**Background:**

Gastroenteropancreatic Neuroendocrine tumors (GEP-NET) are rare neoplasms with limited reported data from the Middle East. Our study aims to report the clinicopathological feature, treatment patterns, and survival outcomes of patients with GEP-NET from our part of the world.

**Methods:**

Medical records of patients diagnosed with GEP-NET between January 2011 and December 2016 at a single center in Saudi Arabia were reviewed retrospectively, and complete clinicopathological and treatment data were collected. Patients’ survival was estimated by the Kaplan–Meier method.

**Results:**

A total of 72 patients were identified with a median age of 51 years (range 27–82) and male-to-female ratio of (1.1). The most common tumor location was the pancreas (29.1%), followed by small bowel (25%), stomach (12.5%), rectum (8.3%), colon (8.3%), and appendix (6.9%). Forty-one patients (57%) had well-differentiated grade (G)1, 21 (29%) had G2, and 4 (6%) had G3. In five patients, the pathology was neuroendocrine carcinoma and in one it could not be classified. 54.2% of the patients were metastatic at diagnosis. Forty-two patients underwent surgical resection as primary management while 26 underwent systemic therapy, three patients were put on active surveillance, and one was treated endoscopically with polypectomy. The 5-year overall survival and progression-free survivals were 77.2% and 49%, respectively, for the whole group. Patients with G1 and 2 disease, lower Ki-67 index, and surgically treated as primary management had significantly better survival outcomes.

**Conclusion:**

Our study suggests that the most common tumor locations are similar to western reported data. However, there seems to be a higher incidence of metastatic disease at presentation than in the rest of the world.

## Introduction

Neuroendocrine tumors (NET) are heterogeneous tumors that arise from the secretory cells of the diffuse neuroendocrine system throughout the body [1]. They are usually slow-growing tumors and may secrete a variety of peptide hormones. Despite being rare, the incidence has been increasing as reported in the USA, with an incidence of 1.09/100,000 in 1973 to 5.25/100,000 in 2004 [2]. Data from 6 European countries showed an incidence rate of 1–2/100000, with some more predominance in females [3]. No data exist on the incidence from the Middle East. There have been several classifications, one of which represents the embryonic origin. This includes foregut tumors, which arise from the gastroduodenal area, midgut tumors from the jejunum, ileum, and caecum, and hindgut tumors from the distal colon rectum [4]. Gastroenteropancreatic NET (GEP-NET) represent the majority of NET (with an incidence of more than 60%) [5]. The highest incidence has been reported in the rectum and small intestine, followed by the colon, the pancreas, stomach, and appendix [5].

Secretory function accounts for the presenting symptoms of some tumors. Hindgut tumors are rarely secretory, while midgut tumors, mainly when metastatic, often secrete serotonin and other vasoactive substances, causing the typical carcinoid syndrome complex of diarrhea, flushing, and right-sided valvular heart disease [6].

The clinical course of NET has been studied from real-world data. It has become apparent that the tumor's aggressiveness is linked to the site of origin. Small intestine tumors typically have high malignant and metastasis potential but tend to grow slowly. On the other hand, gastric and rectal tumors have a low tendency to metastasize, but when it happens, they rapidly progress [6].

Few data exist on the different types and classes of NET from the Middle East [7]. No consensus statement or guidelines for the management of NET was reported from our region. In this study, we aim to report the clinical presentation, treatment patterns, and outcomes of GEP-NET patients treated at a tertiary care institute in Saudi Arabia.

## Methods

The medical records of all patients diagnosed with gastroenteropancreatic neuroendocrine neoplasms between January 2011 and December 2016 at a tertiary care institution in Saudi Arabia were retrospectively reviewed. Patients were eligible for inclusion in the study if they were ≥ 18 years of age, had histologically confirmed neuroendocrine neoplasm at our institution arising from the pancreas, stomach, small intestine, rectum, colon, or appendix, and received 1^st^ line therapy (including observation) at the same institution. Patients should have had at least one evaluation after starting first-line treatment.

The following data were collected on each patient: demographics, presenting symptoms, date of diagnosis, histological characteristics, primary tumor location, stage at presentation, location of metastasis if any, and imaging modality to establish disease stage (Octreotide scan, Gallium Positron Emission Tomography (Ga-PET), and fluorodeoxyglucose (FDG)-PET). Addationally, the type and date of primary treatment modality, date of recurrence or progression, if any, and status and date of last follow-up. The pathological specimens were reviewed and the diagnosis was reclassified based on the 2017 World Health Organization (WHO) classification [8]. Results of baseline serum chromogranin A level was collected as normal vs. elevated. The test was done as sent out and done by MCR Mayo Clinic Department of Laboratory Medicine & Pathology (200 First St SW Rochester, MN 55,905 Lab Director: Franklin R. Cockerill, III, M.D.).

### Ethical consideration

The research was carried out according to the principles set out in the Declaration of Helsinki 1964 and all subsequent revisions. The study was approved by the research ethics committee of the hospital, which gave the investigator waiver for obtaining informed consent based on the retrospective nature of the study.

### Statistical consideration

Descriptive analyses and frequencies were used to determine patients' characteristics. Progression-free survival was measured from the date of first-line therapy to the date of progression, death, or last follow-up. Overall survival was estimated from the date of first-line treatment to the date of death or last follow-up. Kaplan–Meier survival curves were used to measure survival statistics. The survival outcomes were estimated by Kaplan–Meier survival curves and compared by the Log-Rank test. A *p*-value < 0.05 was considered statistically significant. Statistical analysis was performed using RStudio, version 1.4.1717© 2009–2021 RStudio, PBC.

## Results

**Fig. 1:**
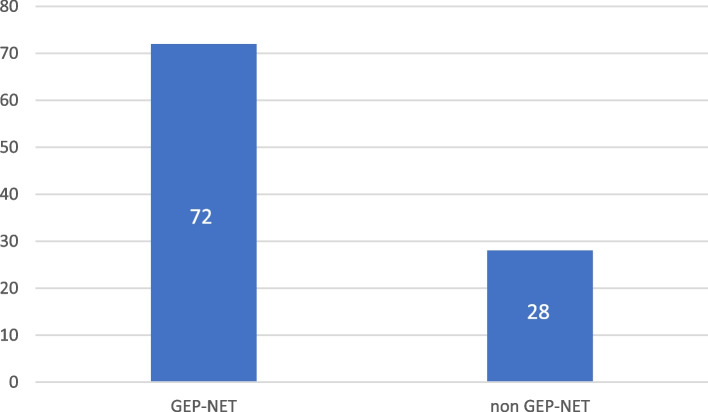
Number of all NET cases identified according to location

Between May 2010 and July 2016, 100 patients diagnosed with NET were identified. Out of those, 72 were GEP-NET. Figure 1 illustrates the number of GEP-NET vs. *non GEP-NET* cases. The characteristics of the 72 patients with GEP-NET are provided in Table 1. Presenting symptoms were as follows: abdominal pain 50 (69%), weight loss 16 (22%), gastrointestinal bleeding 9 (16%), diarrhea 6 (8%), hyperglycemia and flushing in 2 each (3%), and hypoglycemia in 1 (1%). The tumor was discovered incidentally in 8 patients (11%). Primary tumor location was as follows: pancreatic 21 (29.1%), small bowel 18 (25%), stomach 9 (12.5%), rectum and colon 6 each (8.3%), and 5 (6.9%) were appendiceal in origin. Four patients had their tumor in the mesentery, and two in the liver where the primary could not be identified. One patient had the disease in the stomach, duodenum, and pancreas.

**Table 1:** Characteristics of 72 patients with gastroenteropancreatic neuroendocrine tumors

Item	Number (%)
Age	
Median	51
Range	(27–82)
Sex	
Males	38 (52.8)
Females	34 (47.2)
Presenting symptoms	
Incidental	8 (11)
Flushing/diarrhea	7 (10)
Primary tumor location	
Pancreas	21 (29.2)
Other GI^a^ tract	51 (70.8)
Ki-67	
≤ 2%	35 (48.6)
3–20%	23 (31.9)
> 20%	8 (11.1)
Not done	6 (8.3)
Pathology	
Gastrinoma	1 (1.4)
Insulinoma	1 (1.4)
Paraganglioma	1 (1.4)
NET/NEC^b^	69 (95.8)
WHO classification, 2017	
Well-differentiated, G1^c^	41 (57)
Well-differentiated, G2	21 (29)
Well-differentiated, G3	4 (6)
Neuroendocrine carcinoma	5 (7)
Unknown	1 (1)
Disease stage	
Localized	33 (45.8)
Metastatic	39 (54.2)
Metastatic organ involvement	
Lung	11 (15.3)
Liver	31 (43.1)
Peritoneum	8 (11.1)
Lymph nodes	20 (27.8)
Bone	6 (8.3)
Alkaline phosphatase	
Normal	48 (66.7)
Elevated	23 (31.9)
Not done	1 (1.4)
Chromogranin A level	
Normal	11 (15.3)
Elevated	13 (18.1)
Not done	48 (66.7)

^a^ *GI* Gastrointestinal, ^b^ *NET* Neuroendocrine tumor, *NEC* Neuroendocrine carcinoma, ^c^ *G* Grade

Interestingly more than 50% of the patients (39, 54.2%) presented with metastatic disease, with liver metastasis occurring in 30 of them (77%).

Serum chromogranin A was done in 24 patients and was elevated in 13. 24-h urine collection for 5-Hydroxy-Indoleamine acetic acid (5-HIAA) was performed in 26 patients and was elevated in 19 of them. Nine patients underwent Ga-PET, all of which showed uptake in the concerned tumor. In contrast, 19 underwent an Octreotide scan, of which 15 had a positive scan and four were negative. Twenty-three patients had FDG-PET scans done, 8 had no FDG uptake, and 15 had positive uptake. Of the eight negative results, 5 had simultaneous octreotide or Ga-PET scan, and 3 had positive uptake. On the other hand, all 6 of the positive FDG-PET scans with concurrent octreotide or Ga-PET scan were positive.

Of the 72 patients, 42 underwent surgical resection as primary management while 26 underwent systemic therapy, three patients were put on active surveillance, and one was treated endoscopically with polypectomy. The resection margin for those who underwent surgery was negative in 31, positive microscopic margin in 3, residual macroscopic disease in 7, and the margin could not be identified in one.

Systemic therapy consisted of long-acting octreotide in 14, chemotherapy in 8, everolimus in 2, and sunitinib in 1. Commonly used chemotherapy agents were cisplatin, etoposide, temozolomide, and capecitabine. Of the 26 patients receiving systemic therapy at presentation, 12 (46%) patients received second-line systemic therapy, while 5 (19.2%) patients underwent salvage surgery.

Twenty-four patients developed disease recurrence/progression, with two patients upstaged during surgery to metastatic disease. Subsequent treatment was given to 24 patients, including surgery in 3, regional therapy (transarterial chemoembolization) in 2, peptide receptor radionuclide therapy (PRRT) with ^177^ lutetium in 5, and systemic therapy in 14.

**Fig. 2:**
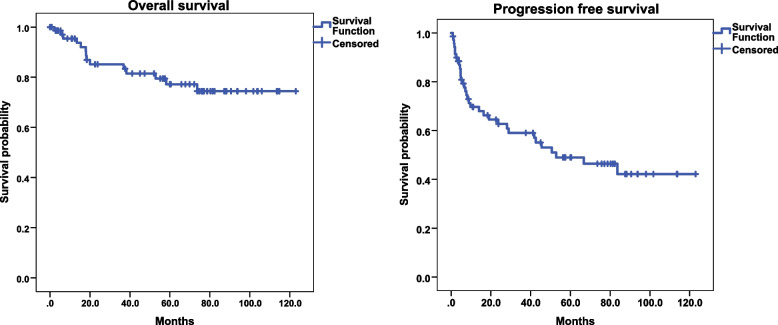
Kaplan Meier blot for overall survival and progression free survival of all patients with GEP-NET

With a median follow-up of 72.9 months, the progression-free and overall survival rate (PFS and OS) for the whole group at five years were 49% and 77%, respectively (Fig. 2). Univariate analysis for OS was performed and showed a significant difference in favor of tumors with Ki-67 ≤ 20% vs. > 20% (*p* 0.001), well-differentiated grade (G) 1, G2 vs. G3 or neuroendocrine carcinoma (NEC) (*p* < 0.001), and surgical vs. primary medical management (*p* 0.001). Univariate analysis for PFS showed similar benefit for tumors with Ki-67 ≤ 20% vs > 20% (*p* 0.001), well-differentiated G1, G2 vs. G3 or NEC (*p* < 0.001), and surgical vs medical primary management (*p* 0.031) (Table 2). Multivariate analysis for PFS showed significant differences for well-differentiated G3 and NEC vs. G1 (*p*-value < 0.001). Similarly, for overall survival with a *p*-value of 0.002 for well-differentiated G3 and a *p*-value < 0.001 for NEC vs. well-differentiated G1. (Table 3).

**Table 2:** Univariate analysis for OS and PFS for patients with GEP-NET

	5-year OS^a^ (%)	*P*-value^b^	5-year PFS^c^ (%)	*P*-value
All patients	77.2	N/A	49	N/A
Sex		0.48		0.65
Female	78.9		52.7	
Male	75.5		46	
Primary site		0.26		0.11
Pancreas	85.7		65.9	
Non-pancreas	72.8		40	
WHO 2017 classification		< 0.001		< 0.001
Well-differentiated, G1^d^	90		68.9	
Well-differentiated, G2	81.7		36.8	
Well-differentiated, G3	25		0	
Neuroendocrine carcinoma	0		0	
Ki-67 index		< 0.001		< 0.001
≤ 2%	92.2		64.4	
3–20%	77.3		37	
> 20%	14.6		0	
Alkaline phosphatase		0.14		-
Normal	85.1		-	
Elevated	68.2		-	
Primary management		0.001		0.031
Surgery	91.5		55.9	
Medical	48.7		29.4	
Resection status		0.002		0.08
Resection	91.5%		58.4%	
No resection	50.7%		34.6%	

^a^ *OS* Overall survival, ^b^ *p*-value by log-rank test, ^c^ *PFS* Progression-free survival, ^d^ *G* Grade

**Table 3:** Multivariate analysis for OS and PFS for patients with GEP-NET

	5-year OS^a^ HR^b^ (95% CI^c^)	*P*-value^d^	5-year PFS^e^ HR (95% CI)	*P*-value
WHO 2017 classification		< 0.001		< 0.001
Well-differentiated, G1^f^	1		1	
Well-differentiated, G2	0.91 (0.17–5.0)	0.9	2.0 (0.88–4.8)	0.09
Well-differentiated, G3	11.3 (2.5–51.3)	0.002	7.0 (2.4–20.3)	< 0.001
Neuroendocrine carcinoma	40.1 (7.8–206.3)	< 0.001	14.8 (4.1–54.2)	< 0.001

^a^ *OS* Overall survival, ^b^ *HR* Hazard ratio, ^c^ *CI* Confidence interval, ^d^ *p*-value by log-rank test, ^e^PFS Progression-free survival, ^f^ *G* Grade

## Discussion

To our knowledge, the current paper represents the first data about GEP-NET from the Arab world, describing the clinical presentation, pathological characteristics, and survival outcomes. In our study, the median age at diagnosis was 51, ranging between (27–82) years. The male-to-female ratio was 1.1 (38:34) more in males similar to reports from other countries [7, 9–11]. Eleven percent of the patients were diagnosed incidentally either by imaging or after surgical resection for other indications. Ten percent of patients had carcinoid symptoms with flushing or diarrhea at presentation, comparable to other reports [12]. Three (4.2%) patients had one of each: gastrinoma, insulinoma, and paraganglioma. The percentage of functional NET in our study is comparable to other studies [9, 13].

Fifty-four percent (54.2%) of our patients were metastatic at diagnosis. The percentage of patients diagnosed with metastasis at presentation is considerably higher than reported incidence from other Middle Eastern countries, East Asia, and the western hemisphere [7, 11–15]. This is likely because NET is slowly growing tumors and tend to present with nonspecific symptoms causing a delay in diagnosis. Additionally, since our institution is a tertiary referral center for cancer, more advanced cases are referred than localized ones. However, reports from East Asian countries have described more localized diseases than other Western countries [5, 9–13]. One hypothesis is that rectal NET is the most common primary site in Asians, and they tend to present earlier with symptoms leading to early diagnosis [5, 9, 10, 16]. The most common primary tumor site in our study was the pancreas, followed by the small bowel. This is in concordance with the western countries' reports rather than Asian reports, where the rectum was the most common primary site [5, 9–13].

We have used in our study the 2017 WHO classification for NET pathology characterization. Most of our patients were well-differentiated, with 57% having G1 and 29% having G2, 6% G3, and 7% NEC. This is consistent with other reports from different parts of the world [9, 13, 16]. Ki-67 in our patients for those with ≤ 2%, 3–20%, and > 20% was 48.6%, 31.9%, and 11%, respectively. Elevated ALP has been described as a poor prognostic factor for NET that predicts shorter survival [17]. However, our patients with elevated ALP had a shorter 5-year OS, but it did not reach statistical significance.

The 5-year survival for our patient's cohort was 77.2%, and the median OS was not reached at the time of data analysis. Overall survival at five years for both males and females was comparable (78.9% for males vs. 75.5% for females, p = 0.488); this interestingly is different from other reports which showed a better survival for females [5]. Others have reported better survival in males [12]. As anticipated, patients who underwent surgical resection as part of their management did better than patients with only medical management with 5-yr OS 91.5% vs. 48.7% and a significantly better PFS. This can be explained as most patients who had surgery likely had localized tumors. Interestingly, it has been shown that even patients with metastatic NET who undergo palliative surgery enjoy better survival than those who don't [12, 13].

Pancreatic primary vs. other gastrointestinal (GI) primaries of GEP-NET did not significantly influence survival in our study, similar to other reports in the literature [12]. A study assessed the survival of patients with NET according to the volume of treating centers using the Surveillance, Epidemiology, and End Results (SEER) registry, suggesting that patients treated in high-volume centers tend to do better with improved overall survival [18].

Five-year survival was significantly lower in patients with well-differentiated G3 NET and NEC than in the well-differentiated G1 and 2 NET (25% in G3, 0% in NEC, 90% in G1, and 81.7% in G2). This is supported by several studies that showed the poor prognosis of patients with poorly differentiated tumors. In our study, all patients with well-differentiated G3 NET and NEC progressed during the follow-up period.

Ga-PET has been shown to be sensitive imaging to identify somatostatin receptor-positive NET and helps in staging and primary tumor site localization for NET of unknown primary [19]. Furthermore, Ga-PET seems to distinguish G3 from G1/2 by assessing the mean standardized uptake value (SUV) [19]. Despite small numbers, our study supports Ga-PET's improved sensitivity and specificity compared to octreotide scan or FDG-PET scan. Of the nine patients who had Ga-PET, all had positive uptake in the primary tumor, while 15 of 19 patients with octreotide scan had uptake in their primary tumor. On the other hand, 8 of 23 patients who had FDG-PET had no uptake in their scan.

Of 26 patients in our study undergoing primary medical therapy, 12 received second-line systemic therapy, while 5 underwent salvage therapy. Others have reported 55% second-line therapy and 31% third-line therapy in patients with metastatic GEP-NET [20].

Our study had some limitations. First, the study's retrospective nature. The second is a single-center study, which took place in a tertiary referral centre which cannot represent the entire country or region. Third, the rather small number of patients.

## Conclusion

In conclusion, our study highlights the frequencies, characteristics, treatment patterns, and results for patients with GEP-NET in our region. The primary site was more in concordance with western data. The higher incidence of patients presenting with metastatic disease indicates the need for better education for healthcare professionals for earlier diagnosis. Treatment patterns and results proved to be similar to other published literature.

## Data Availability

All data and documents needed will be provided upon request through email: bazarbashi@gmail.com.

## Abbreviations

GEP-NET: Gastroenteropancreatic Neuroendocrine tumors
NET: Neuroendocrine tumors
NEC: Neuroendocrine carcinoma
Ga-PET: Gallium Positron Emission Tomography
FDG: Fluorodeoxyglucose
WHO: World Health Organization
5-HIAA: 5-Hydroxy-Indoleamine acetic acid
PRRT: Peptide receptor radionuclide therapy
PFS: Progression-free survival
OS: Overall survival
G: Grade
SUV: Standardized uptake value
GI: Gastrointestinal
NOS: Not otherwise specified
HR: Hazard ratio
CI: Confidence interval
